# Associations of socioeconomic factors with cause-specific Mortality and burden of cardiovascular diseases: findings from the vital registration in urban Shanghai, China, during 1974–2015

**DOI:** 10.1186/s12889-020-09390-1

**Published:** 2020-08-26

**Authors:** Lijuan Zhang, Qi Li, Xue Han, Shuo Wang, Peng Li, Yibo Ding, Tao Zhang, Jia Zhao, Yifan Chen, Jiluo Liu, Jue Li, Xiaojie Tan, Wenbin Liu, Rong Zhang, Guangwen Cao

**Affiliations:** 1 Shanghai East Hospital, Key Laboratory of Arrhythmias, Ministry of Education, Tongji University School of Medicine, Tongji University, Shanghai, 200120 China; 2 Division of Chronic Diseases, Center for Disease Control and Prevention of Yangpu District, Shanghai, China; 3 Division of Chronic Diseases, Center for Disease Control and Prevention of Hongkou District, Shanghai, China; 4 Department of Epidemiology, Second Military Medical University, 800 Xiangyin Rd., Yangpu District, Shanghai, 200433 People’s Republic of China

**Keywords:** Cardiovascular disease, Mortality, Medical insurance, Burden, Malnutrition, Lifestyle, Pollution control, Sociodemographic index, Low- and middle-income countries

## Abstract

**Background:**

Cardiovascular disease (CVD) is the leading cause of mortality worldwide. The effect of socioeconomic factors on cause-specific mortality and burden of CVD is rarely evaluated in low- and middle-income countries, especially in a rapidly changing society.

**Methods:**

Original data were derived from the vital registration system in Yangpu, a representative, population-stable district of urban Shanghai, China, during 1974–2015. Temporal trends for the mortality rates and burden of CVD during 1974–2015 were evaluated using Joinpoint Regression Software. The burden was evaluated using age-standardized person years of life loss per 100,000 persons (SPYLLs). Age-sex-specific CVD mortality rates were predicted by using age-period-cohort Poisson regression model.

**Results:**

A total of 101,822 CVD death occurred during 1974–2015, accounting for 36.95% of total death. Hemorrhagic stroke, ischemic heart disease, and ischemic stroke were the 3 leading causes of CVD death. The age-standardized CVD mortality decreased from 144.5/100,000 to 100.7/100,000 in the residents (average annual percentage change [AAPC] -1.0, 95% confidence interval [CI] -1.7 to − 0.2), which was mainly contributed by women (AAPC -1.3, 95% CI − 2.0 to − 0.7), not by men. Hemorrhagic stroke, the major CVD death in the mid-aged population, decreased dramatically after 1991. The crude mortality of ischemic heart disease kept increasing but its age-adjusted mortality decreased continually after 1997. SPYLLs of CVD death increased from 1974 to 1986 (AAPC 2.1, 95% CI 0.4 to 3.8) and decreased after 1986 (AAPC 1.8, 95% CI − 2.3 to − 1.3). These changes were in concert with the implementation of policies including extended medical insurance coverage, pollution control, active prophylaxis of CVD including lifestyle promotion, and national health programs. The mortality of CVD increased in those born during 1937–1945, a period of the Japanese military occupation, and during 1958–1965, a period including the Chinese Famine. Sequelae of CVD and ischemic heart disease are predicted to be the leading causes of CVD death in 2029.

**Conclusions:**

Exposure to serious malnutrition in early life might increase CVD mortality in later life. Improvements in medical services, pollution control, and lifestyle could decrease CVD death. New strategy is needed to prevent the aging-related CVD death and burden in the future.

## Background

Although cardiovascular disease (CVD) is preventable and treatable, the number of global CVD death has increased by 12.5% during the last decade. CVD accounts for one third of global human death (17.7/55 millions) in 2017 [1, 2]. In the Western world, the mortality rate of CVD has decreased since the mid-1990s due to improvements in acute care and the efficacy of secondary prevention [3]. In low- and middle-income countries (LMICs), a substantial number of CVD death are attributed to tobacco abuse, physical inactivity, unhealthy diet, harmful alcohol use, air pollution, poor food supply, low education level, uncontrolled blood pressure, and psychosocial aspects [4–7]. China is one of the largest LMICs. Population in mainland China experienced dramatic sufferings and alterations after the First Opium War (1840–1842). Since the founding of the People’s Republic of China in 1949, several demographic and socioeconomic challenges accompanied by social development have been reported: a rapid population swell during 1952–1982 [8], the rapid economic expansion since 1978 [9], life expectancy increases since 1981 [10], aging society since 1999 [11], unhealthy lifestyle since 1990 [9], environmental pollution [12, 13], and increases in conventional CVD risk factors such as hypertension, dyslipidemia, diabetes mellitus, overweight, and obesity [14–17]. Thereafter, specific steps and responses were taken to meet these challenges including a series of programs to promote healthy lifestyles targeting to the risk factors of CVD, improvement of primary care since 1997, basic medical insurance coverage in 2008, hospital care, and public health actions such as air pollution control action plan [9]. However, the mortality rate of CVD has increased significantly during the last 2 decades and CVD is still the top cause of human death in China [18]. There is a large gap between medical/public health actions and effective prophylaxis of CVD in China. Perinatal and postnatal exposure to indoor or outdoor air pollution and other adverse events contributes to the health (allergic and infectious diseases) in children, especially in LMICs [19–22]. However, its contribution to the mortality of CVD remains to be clarified. The influence of socioeconomic factors including early life exposure to adverse events, economic activity, atmosphere pollution, income per capita, and education and national health promotion plans on the CVD-caused immature death has not been systemically evaluated.

As the economic center in China, Shanghai belonged to low-income region before 1994, lower-middle income region between 1996 and 2005, and upper-middle income region after 2006 [23]. Shanghai stands at the forefront of the national strategic initiatives and responds quickly to the economic reform and health promotion policies. Therefore, Shanghai is one of the most suitable places to clarify the effects of socioeconomic changes and national health promotion actions on CVD death in China. We selected Yangpu district of Shanghai as a study district based on the following reasons. First, pollution-causing industries including coal-fired power generation, textiles, steel metallurgy, electroplating, and coal gas production had been introduced since the old municipal government established in Yangpu in 1933 and were shut down in the 1980s. Second, the permanent residents in Yangpu, rather than in entire Shanghai, have been quite stable during 1974–2015, without immigration influx or efflux. Effects of changing risk factor exposures on the causes of CVD deaths should be only evaluated in a stable population. The epidemiological transition in CVD mortality occurred earlier and slower in western countries, but it has been compressed into a few decades in China, that is, the main cause of population death has shifted from infectious diseases and perinatal diseases to chronic diseases [24]. Third, the vital registration system in Yangpu was established 14 years prior to the national one established in 1987 [25].

In the present study, we characterized long-term trends in the mortality and burden of CVD in Yangpu, a representative district of urban Shanghai, to evaluate the influence of socioeconomic events and health actions on cause-specific CVD death during 1974 and 2015. This study may help in improving CVD control strategy in other countries, especially in LMICs.

## Methods

### Data sources

Information on CVD death during 1974–2015 was derived from the vital registration system, covering the all registered residents of Yangpu district, Shanghai [25]. The details of each CVD patient including age, gender, date, and cause of death were collected. The majority (97.94%) of CVD death were diagnosed with solid clinical and laboratory evidence or morphologic verification. Death from each CVD cause during 1974–2001 was classified based on the International Classification of Disease-9th version (ICD-9) codes. Since 2002, causes of CVD death were classified according to the ICD-10 codes (Table S1 in Additional file). Causes of CVD death were estimated for a comprehensive category that combined CVD and circulatory conditions. These causes were ischemic heart disease (IHD), hemorrhagic stroke (HS), ischemic stroke (IS), hypertensive disease (HD), rheumatic heart disease (RHD), sequelae of cerebrovascular disease (SCD), other forms of heart diseases (heart failure, pericarditis and other diseases of pericardium, acute and subacute endocarditis, heart valve diseases, myocarditis and cardiomyopathy, conduction diseases, cardiac arrest, cardiac arrhythmias), and other cardiovascular and circulatory diseases. For stroke death estimates, the GBD defined stroke ICD codes as HS, IS or non-specific as to type, non-specific codes were redistributed to HS or IS using a regression model [26]. The study was performed in accordance with the 2000 Declaration of Helsinki and was approved by the ethics committee of Second Military Medical University.

### Estimation of CVD Mortality and burden

The mortality rates from 1974 to 2015 stratified by sexes and eighteen 5-year age groups were calculated. All the rates were calculated as per 100,000 persons per year. Age standardized mortality rates were calculated using Segi’s world standard population [25, 27]. Age-standardized mortality and birth cohort were applied to characterize the temporal trends of CVD mortality. Person years of life lost (PYLL), age-standardized person years of life loss per 100,000 persons (SPYLLs) and average years of life lost (AYLL) were applied to estimate CVD burden. All analyses were performed separately by sex and aggregated by 5-year age categories. Burden of all-cardiovascular and each underlying CVD cause were estimated using PYLL, SPYLLs and AYLL. Formula and calculation for disease burden were as follows:

$$ \mathrm{PYLL}=\sum \limits_{i=1}^e{a}_i{d}_i $$

$$ \mathrm{SPYLLs}=\frac{\mathrm{PYLL}\times 100\ 000}{n} $$

$$ \mathrm{AYLL}=\frac{\mathrm{PYLL}}{\sum {d}_i} $$

e = the life expectancy in Shanghai (Table S2 in Additional file); *i* = median age of the death group; *a*_*i*_ = e-(i + 0.5); *d*_*i*_ = number of deaths at age; *n* = general population.

### Sociodemographic index

Sociodemographic Index (SDI) was applied to examine changes in the major causes of CVD death burden as a function of epidemiological transition. SDI was estimated using equally weighted age-sex-state-year-specific geometric means of lag-dependent income per capita, average educational attainment in the population over age 15 years, and the total fertility rate, as previously described [28].

### Statistical analysis

Temporal trend in the mortality of CVD could be theoretically attributed to aging, characterized by changes in cumulative exposure to risk factors over time (age effects), changes occurring during specific calendar periods irrespective of age (a period effect), or changes affecting persons born in specific or successive generations irrespective of age (a cohort effect). An age-period-cohort (APC) model was fitted to better understand the effects of the three factors on disease rates [29]. Segmented package in R software (Version 3.3.3) was applied to conduct the APC model [25]. Leslie matrices were applied to predict cause of CVD death cases and person-years by age (a), period (p) and cohort (c) on mortality and predict mortality trend from 2015 to 2029 [30]. Temporal trends for the crude, log-transformed and age-standardized mortality rates and the burden of CVD from 1974 to 2015 were carried out using Joinpoint Regression Software, version 4.7.0.0, provided by the Surveillance Research Program of the National Cancer Institute (Bethesda, MD). The trend was expressed as an average annual percentage change (AAPC). The z test was employed to assess whether APC was statistically different from zero; 95% confidence interval (95% CI) for each segment was calculated. Other statistical analyses were conducted using SPSS 23.0 (SPSS, Chicago, IL).

## Results

### General information

All registered permanent residents in Yangpu, with a total of 41,879,864 person-years, were analyzed. A total of 101,822 CVD deaths (51,423 women vs. 50,399 men) occurred, accounting for 36.95% of all death. Age-standardized mortality rate for all CVD was higher in men than in women (155.2/10^5^ vs.121.4/10^5^, *P* = 4.6 × 10^− 6^). IHD, HS and IS and SCD were the 4 leading causes of CVD deaths in both sexes (Fig. S1 in Additional file), accounting for 80.16% in crude mortality and 82.01% in age-standardized mortality for all CVD.

### Composition of CVD death

**Fig. 1:**
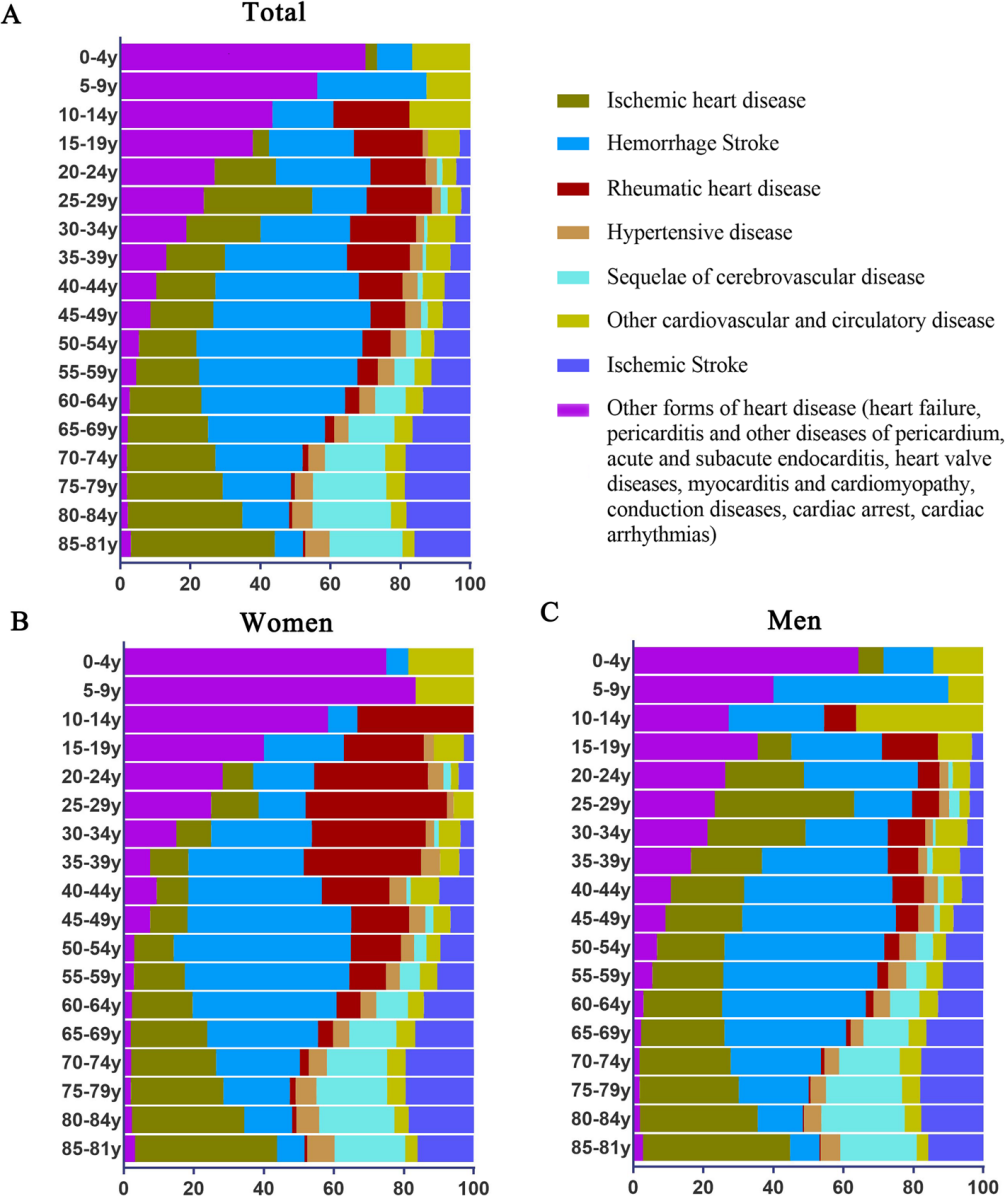
Proportion of age-standardized mortality rates per 100,000 persons for the major causes of CVD in Yangpu, Shanghai, China, 1974–2015

IHD death and IS death increased with aging and SCD death increased after 50 years in all populations (Fig. 1a); RHD was the main cause of CVD deaths in women within 10–39 years (Fig. 1b); HS was the leading cause of CVD deaths at 35–69 years in both women and men (Fig. 1b and c).

### Trends in CVD death

**Fig. 2:**
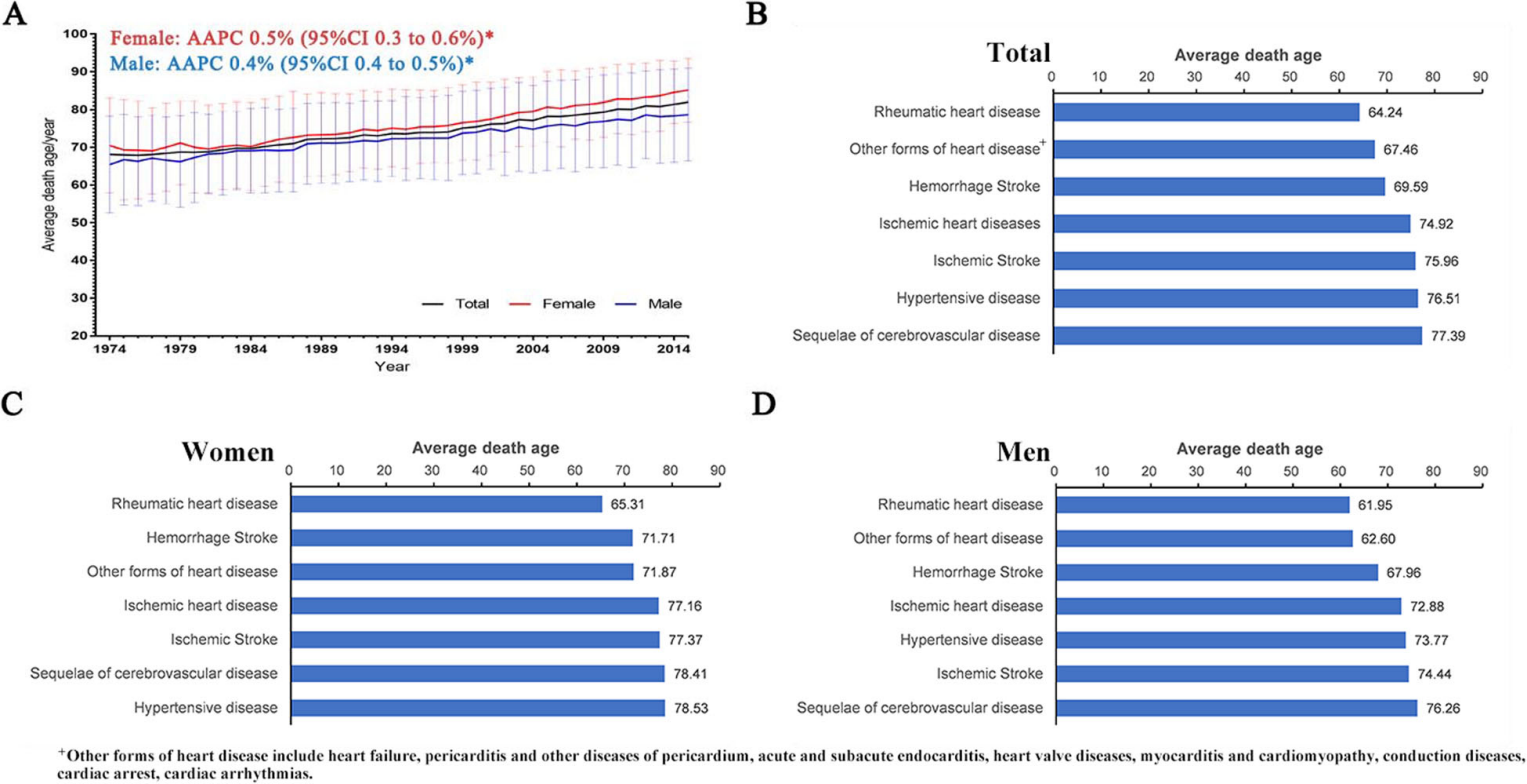
Average age of the residents died of the major causes of CVD in Yangpu district, Shanghai, China, 1974–2015. **Note:** AAPC, average annual percentage change

Average age of CVD death was 68.13 ± 12.93 years in 1974 and increased consecutively during 1974–2015 (AAPC 0.5, 95% CI 0.3 to 0.6 in women; AAPC 0.4, 95% CI, 0.4 to 0.5 in men) (Fig. 2a). Men died of CVD earlier than did women (72.26 ± 11.41 vs. 75.75 ± 10.45 years, *P* = 0.001). RHD was the top CVD type that caused earlier death in both men and women (Fig. 2b-d).

**Fig. 3:**
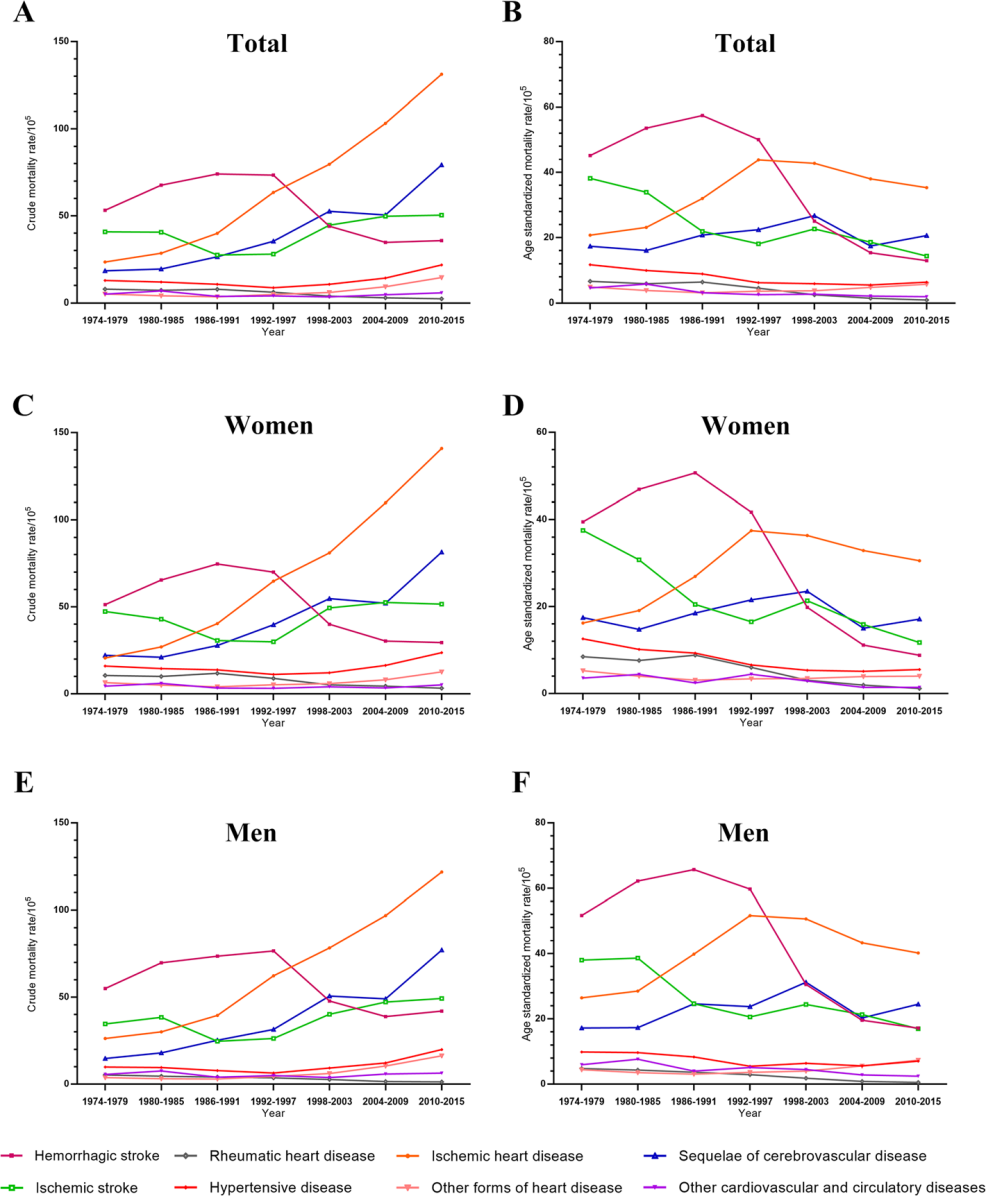
Trends in crude and age-standardized mortality rates of the major causes of CVD in both sexes in Yangpu, Shanghai, China, 1974–2015. **Note: a** trend in crude mortality rates in all residents; **b** trend in age-standardized mortality rates in all residents; **c** trend in crude mortality rates in women; **d** trend in age-standardized mortality rates in women; **e** trend in crude mortality rates in men; **f** trend in age-standardized mortality rates in men

The age-standardized mortality rate of total CVD decreased in all the residents (AAPC -1.0, 95% CI − 1.7 to − 0.2) and in women (AAPC -1.3, 95% CI − 2.0 to − 0.7) during 1974–2015; however, it did not alter in men (AAPC -0.7, 95% CI − 1.5 to 0.1) (Table S3 in Additional file). The crude mortality rate of IHD consecutively increased whereas the rate of HS decreased sharply after 1997. The crude mortality curves of HS and IHD, the two major CVD types, converged at 1997–1998 (Fig. 3a, c, e). The age-standardized mortality rate of HS increased during 1974–1991 (AAPC 0.9, 95% CI 0.1 to 1.7) and then decreased dramatically during 1991–2003 (AAPC -14.5, 95% CI − 18.1 to − 10.8); the age-standardized mortality rate of IHD increased dramatically during 1984–1997 and then decreased (AAPC -1.6, 95% CI − 2.1 to − 1.1). The age-standardized mortality curves of HS and IHD also converged at 1997–1998 (Fig. 3b, d, f). The age-standardized mortality rates of IS and RHD kept decreasing during 1974–2015 (AAPC -2.5, 95% CI − 3.1 to − 1.8 and AAPC -6.0, 95% CI − 7.1 to − 4.9, respectively) (Table S3 in Additional file).

### Age, period and cohort effects on the main causes of CVD death

**Fig. 4:**
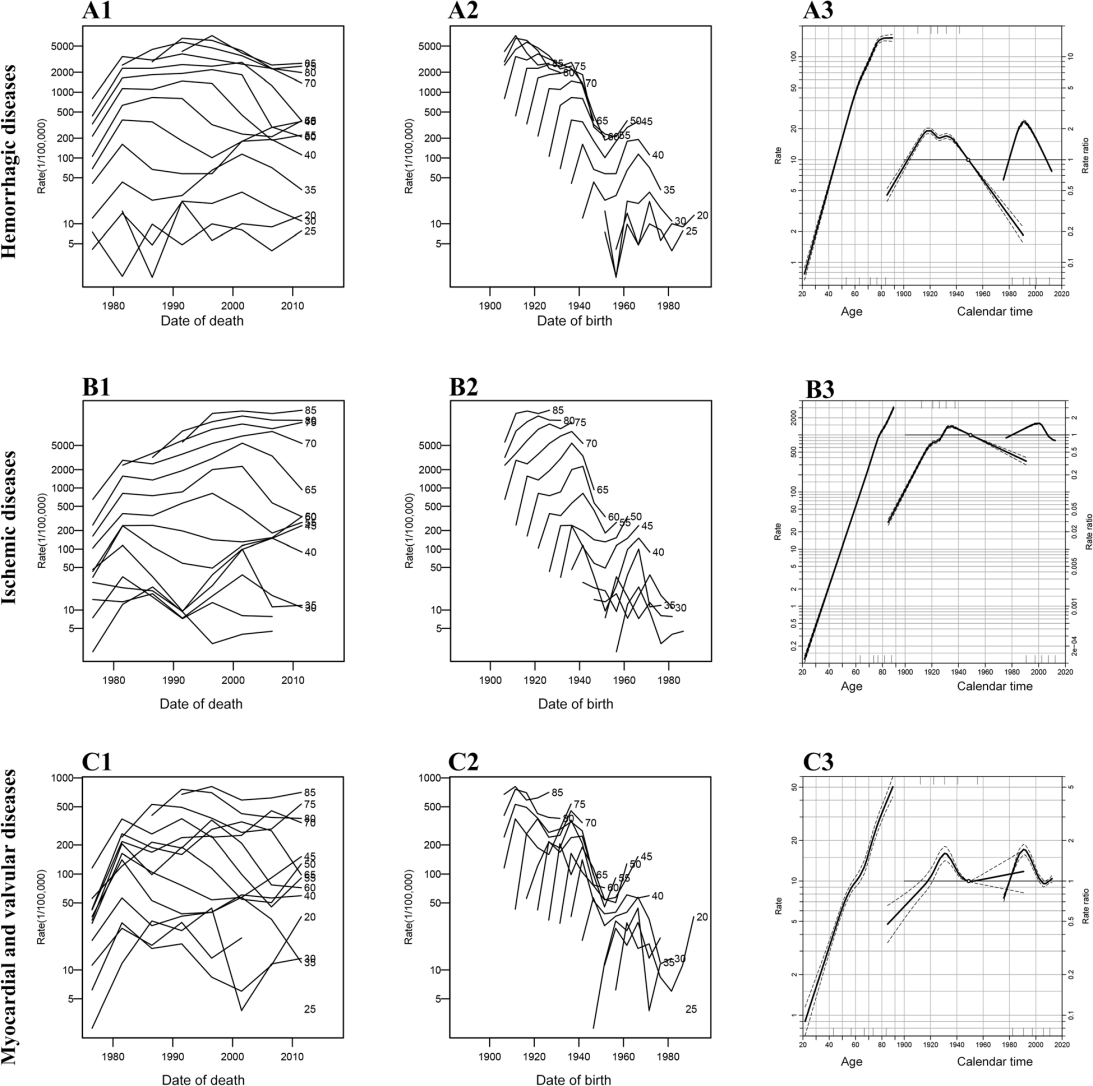
Age-specific mortality rates (per 100,000) by period and birth cohort and age, period, and cohort effects for the mortality rates of the major CVD in Yangpu, Shanghai, China, 1974–2015. **Note:** Each row of plots, from left to right, are age-specific mortality rates by period, age-specific mortality by birth cohort, and an age-period-cohort Poisson (APC) regression plot. The APC regression plot has 3 curves depicting, from left to right, trends in mortality rate by age (yr) for the reference birth cohort (1949), the risk ratio of the cohort effect compared with the reference birth cohort (1949), and the risk ratio of the calendar period effect compared with the reference cohort (1980). Dotted lines show the 95% confidence intervals of the 3 components (solid lines)

We reclassified the causes of CVD into hemorrhagic disease (HS and HD) and ischemic disease (IHD and IS), and myocardial valvular diseases (RHD, other forms of heart disease, and other cardiovascular and circulatory diseases). Deaths from hemorrhagic disease increased sharply during 1974–1982, increased slightly during 1982–1992, and greatly decreased after 1992–2000 in the age groups of 50–70 years (Fig. 4A1). Deaths from ischemic disease increased consecutively in the age groups above 70 years (Fig. 4 B1). Birth cohort analysis indicated that deaths from all CVD increased in population born during 1937–1945 and increased again in those born during 1958–1965 (Fig. 4A2, B2, C2). The fitting trend of the birth cohort indicated that the risks of death from hemorrhagic disease increased for 4 fold in the population born during 1885–1920, and then decreased sharply in the population born after 1935 (Fig. 4A3); whereas the risks of death from ischemic disease increased for 33 fold in the population born during 1885–1935, and then decreased slightly in the population born after 1935 (Fig. 4B3). The data of myocardial valvular diseases were in a jumble, indicating the heterogenicity in their etiology (Fig. 4C3).

### Association of SDI with the main causes of CVD death

With an increase in SDI, the age-standardized mortality for all CVD declined during the study period and the regression coefficient (*β*) was − 0.690 (*P* = 4.3 × 10^− 7^). RHD and hemorrhagic disease gradually declined with an increase of SDI during 1974–2015, with the *β* value of − 0.927 for RHD, − 0.796 for HS, and − 0.781 for HD, respectively (all *P <* 0.001). The same was true for IS (*β* = − 0.684*, P* = 5.9 × 10^− 7^). However, the mortality rate of IHD increased with increasing SDI (*β* = 0.646*, P* = 4.0 × 10^− 5^). Other causes of CVD deaths did not display an association with the change of SDI (all *P* > 0.05) (Table S4 in Additional file).

### Burden of CVD death during 1974–2015

**Fig. 5:**
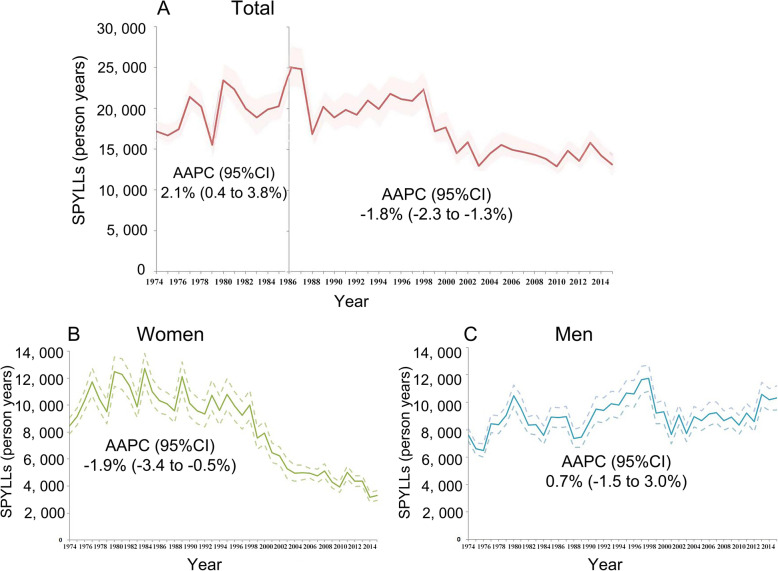
Trend in age-standardized CVD person years of life lost (SPYLLs) per 100,000 persons in Yangpu district, Shanghai, China, 1974–2015. **Note:** Solid lines, mean value; dotted lines, standard error

SPYLLs of CVD death were 17,187 person years in 1974 and increased in the following 12 years (AAPC 2.1, 95% CI 0.4 to 3.8), then decreased at an annual average rate of 1.8 (95% CI − 2.3 to − 1.3) during 1986–2015 (Fig. 5a). This reduction mainly came from women (Fig. 5b), not from men (Fig. 5c). Using the Shanghai life table as a reference, AYLL were 13.4 years for CVD death. AYLL did not change over time, with a mean change of 0.2 year (95% CI − 0.1 to 0.4).

**Fig. 6:**
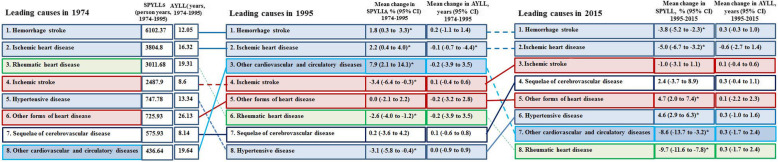
Changes in SPYLLs and AYLL for the major causes of CVD in Yangpu, Shanghai, China. **Note:** Solid lines are “increases” and dashed lines are “decreases”. For the time period 1974–1995 and 1995–2015, two measures of change are shown: percent change in SPYLLs, change in SPYLLs, change in AYLL. Statistically significant changes are shown with *. SPYLLs = age-standardized person years of life lost per 100,000 persons. AYLL = average years of life lost

PYLLs for IHD kept increasing (Table S5 in Additional file), whereas SPYLLs for IHD kept stable during 1974–2015 (Table 1), indicating aging is the major determinant. SPYLLs for HS increased before 1994 and then decreased rapidly during 1994–2005. However, HS and IHD remained the first 2 causes for SPYLLs and AYLL of CVD in 1974 and subsequent two 20 years (1974–1995 and 1995–2015). RHD was one of the 3 leading causes for SPYLLs and AYLL in 1974, but fell by annual average percent of − 2.6 (95% CI − 4.0 to − 1.2) during 1974–1995 before rapidly falling off after 1995. IS became the 3rd leading cause for SPYLLs during 1995–2015. The annual average percent of SPYLLs for other forms of heart disease increased after 1982 (AAPC 4.2, 95% CI 3.0 to 5.4) (Fig. 6**).**

**Table 1:** Trends in SPYLLs for the major types of CVD in Yangpu, Shanghai, China, 1974–2015

Types of CVD	AAPC (95% CI) (1974-2015)	Joinpoint trend 1		Joinpoint trend 2		Joinpoint trend 3	
		Years	AAPC (95% CI)	Years	AAPC (95% CI)	Years	AAPC (95% CI)
Ischemic heart disease	0.3 (-0.1 to 0.8)						
Hemorrhagic stroke	-1.3 (-2.4 to -0.2) ^b^	1974-1994	1.5 (0.4 to 2.6) ^b^	1994-2005	-8.7 (-11.2 to -6.1) ^b^	2005-2015	1.7 (-1.0 to 4.4)
Ischemic stroke	-0.3 (-1.3 to 0.6)						
Sequelae of cerebrovascular disease	3.1 (-4.3 to 10.8)	1974-1977	66.1 (6.1 to 160.1) ^b^	1977-1980	-24.5 (-69.8 to 89.1)	1980-2015	1.6 (0.5 to 2.7) ^b^
Hypertensive disease	1.0 (-1.2 to 3.2)	1974-1977	14.4 (-12.3 to 49.3)	1977-1992	-6.0 (-8.2 to -3.7) ^b^	1992-2015	4.6 (3.3 to 6.0) ^b^
Rheumatic heart disease	-6.5 (-8.2 to -4.8) ^b^	1974-1979	-10.7 (-19.0 to -1.5) ^b^	1979-1991	1.2 (-2.3 to 4.8)	1991-2015	-9.2 (-10.8 to -7.6) ^b^
Other forms of heart disease^a^	1.8 (-0.7 to 4.3)	1974-1982	-7.8 (-18.3 to 4.1)	1982-2015	4.2 (3.0 to 5.4) ^b^		
Other cardiovascular and circulatory diseases	-1.0 (-12.0 to 11.4)	1974-1990	0.4 (-6.2 to 7.4)	1990-1993	64.4 (-66.8 to 715.3)	1993-2015	-8.5 (-12.0 to -4.9) ^b^

*AAPC* average annual percentage change, *CVD* cardiovascular disease, *95% CI* 95% confidence intervals, *SPYLLs* age-standardized person years of life lost per 100,000 persons

^a^Other forms of heart disease include heart failure, pericarditis and other diseases of pericardium, acute and subacute endocarditis, heart valve diseases, myocarditis and cardiomyopathy, conduction diseases, cardiac arrest, cardiac arrhythmias

^b^AAPC value is significantly different from 0 at α=0.05

**Fig. 7:**
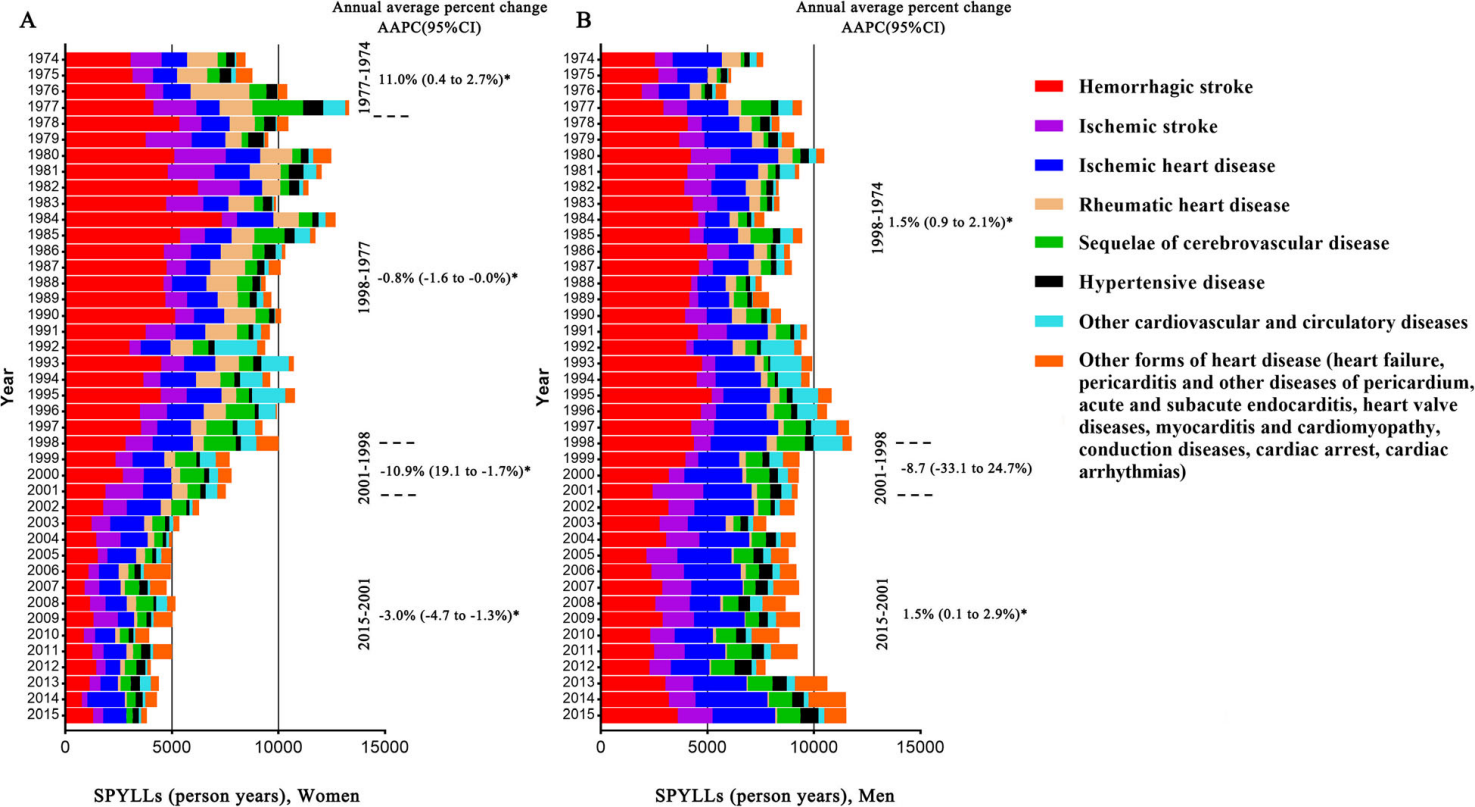
Trends in SPYLLs of the major causes of CVD in both sexes in Yangpu district, Shanghai, China, 1974–2015. **Note:** SPYLLs = Age-standardized person years of life lost per 100,000 persons

Total CVD burden decreased over the 42 years in women. The largest annual percentage decrease occurred from 1998 to 2001 (AAPC -10.9, 95% CI − 19.1 to − 1.7), further decreased at an annual average rate of − 3.0 (95% CI − 4.7 to − 1.3) in the following 15 years. In women, HS contributed to 30–50% of CVD SPYLLs before 1998 while HS, IHD and IS became the 3 major sources of CVD SPYLLs after 1998 (Fig. 7a). In men, total CVD burden kept stable during 1974–2015, slightly increased during 1974–1998 and 2001–2015. HS, IHD and IS remained to be the major causes of CVD SPYLLs during 1974–2015 (Fig. 7b).

### Projecting causes of CVD death in 2025–2029

**Fig. 8:**
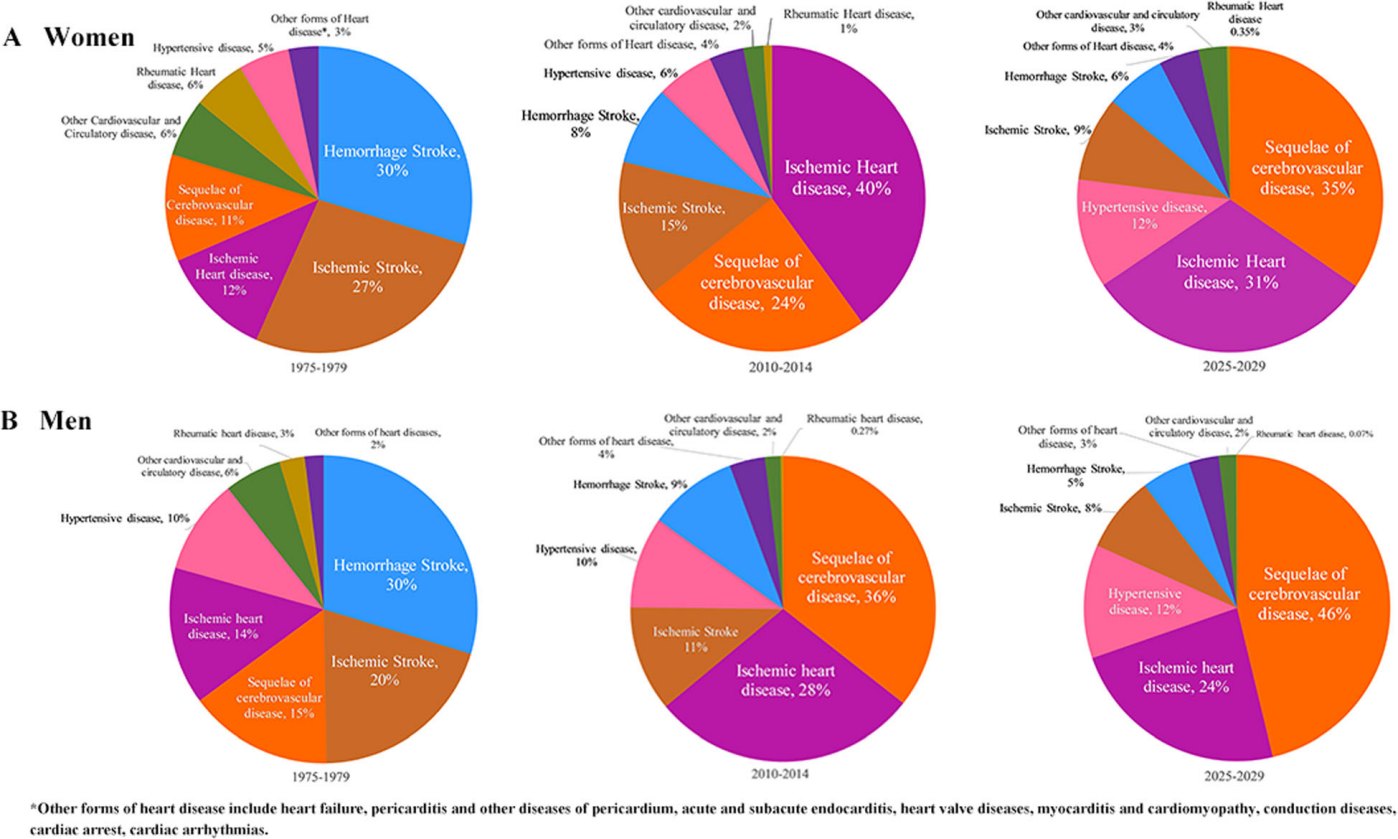
Pie charts of cause-specific CVD death in women and men from 1976 to 1980 and from 2011 to 2015, and predicted from 2025 to 2030, scaled to the number of deaths during each period, (**a**) for women; (**b**) for men

A total of 16,753 women and 22,690 men are predicted to die of CVD during 2020–2029 in the study population. The proportion of SCD kept increasing during 1974–2014 and will be the top cause of CVD death in both sexes during 2025–2029. IHD will be the second leading cause during 2025–2029 in both sexes. HS and IS, the top 2 causes of CVD death during 1975–1979, will drop to the fifth and fourth causes of CVD death during 2025–2029, respectively **(**Fig. 8**)**. RHD accounted for less and less CVD death in the 3 consecutive time periods.

## Discussion

In this study, we evaluated the trends and burden of major CVD death in a rapidly developing society. HS, IHD and IS were the top 3 leading causes of CVD death during 1974–2015. As the leading cause of CVD immature death, HS death mainly occurred among occupational population aged 35–69 years in both sexes. Except for those unmodifiable HS risk factors include age, sex, race-ethnicity and genetics, modifiable risk factors such as hypertension, smoking, waist-to-hip ratio, diet and heavy alcohol consumption were well established [31, 32]. HS is highly prevalent in LMICs where the burdens of hypertensive disorders are heavy [32]. HS accounts for < 30% of stroke death in USA, whereas it accounts for 30–40% of all stroke death in China [33]. Alcohol and smoking consumptions have direct relationships with HS. The consumptions are strongly related to a specific worksite and time of shift, job stressors, and air pollution [34, 35]. Heavy alcohol and smoking consumptions are linked to hypertension and poor blood pressure control in hypertensive patients [36, 37]. Air pollution factors such as nitrogen dioxide and ozone also increase the risk of HS death via increasing the incidence of hypertension [38–40]. HS death started to decrease from 1995, possibly due to 4 reasons: first, hypertension was under the control because anti-hypertensives were covered by medical insurance in China since 1992; second, effective control of blood pressure owing to the World Bank-supported mass health promotion program in 1996 decreased HS-related death; third, pollution-causing industries were removed during the 1980s, which greatly reduced ambient pollution; fourth, medical insurance and medical reform since 1990 have got initial achievements.

IHD, the 2nd cause of CVD-related immature death during 1974–1995, ranked the leading cause of CVD deaths after 1995, which was in accord with that in the whole Chinese population [41]. Marked regional differences in epidemiological trends in age-standardized mortality of IHD have been documented in China. In 2015, the mortality from IHD in Heilongjiang province located in the north east region and Shanghai in the southeast region of China was 187.4 per 100,000 and 44.2 per 100,000, respectively [42]. This difference might be caused by relatively higher SDI, less ambient or indoor pollution in winter, higher temperature, and more advanced health care systems in Shanghai. The crude mortality of IHD kept increasing in both sexes, possibly because of the increase in the proportion of aged population [10]. Ambient pollution and consumption of red and processed meat are positively associated with the risk of IHD, possibly because of high serum non-high-density lipoprotein cholesterol concentration and systolic blood pressure; whereas regular excise like cycling is inversely associated the risk of IHD [42–45]. The age-standardized IHD mortality began to decline slowly after 1998 although population in Shanghai is aging at an unprecedented level since 1995. Improvements in medical service and healthy lifestyle promotion should be effective in decreasing IHD death. IS has been associated with low income, low education levels, hypercholesterolemia, physical inactivity, smoking, and obesity [46–48]. IHD and IS had been recognized as a single type of CVD called ischemic disease in international guidelines because they shared pathologies and risk factors, and shared strategies for primary and secondary prevention [18]. Ischemic disease increased with increased total cholesterol and decreased with elevated high-density lipoprotein cholesterol [49]. The trend in ischemic disease is affected by aging, SDI, lifestyle change, and the quality of medical care. Smoking led to a 63% higher risk of ischemic disease in urban male smokers [50]. A study of the China Kadoorie Biobank has demonstrated that individuals in the top quintiles of physical activity have a 23% lower risk of major ischemic events than those in the bottom quintiles [51]. Compared to people who never or rarely ate fresh fruit, people consuming fresh fruit daily had a 34% lower risk of IHD and a 25% lower risk of IS [52]. Total IHD death had been decreased significantly since the Chinese government issued the “National Physical Fitness Program” in 1995. Pledging sports and health-building services would be aligned with national economic development. Residents in Shanghai kept the habit of daily consumption of fresh vegetables and fruits during the 42 years, the proportion of daily consumption varied from 34 to 39%, which may contribute to changes in CVD mortality curves (Fig. S2 in Additional file). The mechanisms by which fruits and vegetables protected CVD death include not only some known bioactive nutrient effects, but also their functional properties including reducing antioxidant stress, improving plasma lipoproteins, lowering blood pressure, improving insulin sensitivity, and regulating hemostasis [53]. These evidences indicate that ischemic CVD should be prevented via improving lifestyles such as quitting smoking, increasing physical activity, and consuming fresh vegetables and fruits.

RHD, the main cause of CVD deaths in children and young adults in LMICs, results from an abnormal autoimmune response to a group A streptococcal infection [54]. Preventive measures, based on antibiotic treatment especially penicillin use, are very efficient. Thus, RHD reflects a poor socioeconomic condition that lead to lack of medical resources. With steady socioeconomic growth during 1974–2015 in Shanghai, death from RHD kept decreasing rapidly.

In this study, we found that death of hemorrhagic CVD, ischemic CVD, and myocardial valvular CVD increased in population born during 1937–1945 and increased again in those born during 1958–1965. This might be caused by early life exposure to serious adverse condition especially malnutrition during the Japanese military occupation (1937–1945) and the Chinese Famine (1958–1961), respectively. Exposures to adverse living condition and malnutrition in early life are often associated with metabolic syndrome including hyperglycemia, hypertension, and type 2 diabetes that contribute to an increased occurrence of CVD in later life [55–58]. Thus, to decrease adverse events including malnutrition in early life should be important for the prevention of the major types of CVD in later life.

Over the 42-year study period, a reduction in the age-standardized CVD mortality was greater in women than in men. The age-standardized mortality rate and SYPLL of CVD kept decreasing in women after 1977 and declined sharply after 1998 but did not decline anymore in men, which is consistent with a previous observation in China [59]. In the twenty-first century, HS, IHD, and IS have become the 3 main lifetime threats to men and shown a growing trend, suggesting that specific research and health promotion strategies of CVD for men should be brought to attention. This sex disparity is possibly caused by different levels of risk factor exposure. In China, men are more likely to be exposed to factory air pollution, tobacco smoking, and alcohol abuse [60]. Smoking cessation and alcohol restriction should be effective in controlling more CVD-caused immature death in men.

Further, SCD will be the leading cause of CVD during 2025–2029. This suggests that with the improvement of medical services, the first episode of CVD rarely lead to death, and turn into sequelae stage, which will prolong lifetime but may reduce the healthy life year. It also weakens the quality of life and aggravates the burden, hence the urgent need for effective rehabilitation care to prevent disability caused by sequelae of CVD in the future.

This study has major strengths including a stable and large population, a social context of rapid economic development and intensive change of the policies, and stringent mortality ascertainment. However, there are certain limitations to be acknowledged. First, although death registration in Yangpu is one of the high-quality systems in China, the death certification was too precise to assign CVD subtypes using ICD codes, only 7 causes of CVD deaths were demonstrated, other CVD subtypes were grouped as “other cardiovascular and circulatory diseases”. Second, lifestyle, disease history, and medical care data were not included in this system, so it was impossible to quantitate the associations of the risk factors with CVD death.

Conclusively, although the trends in cause-specific mortality and burden of CVD in urban Shanghai have generally declined during 1974–2015, the threat of CVD to human life is still the primary concern. Exposure to adverse event and malnutrition in early life contributed to an increase in CVD death. It is necessary to improve air quality, strengthen health education, advocate smoking cessation, restrict alcohol consumption, and popularize reasonable diet. Medical insurance and improvement in medical service are important for the prevention and control of CVD death. Effective rehabilitation is needed to prevent the disability caused by sequelae of CVD.

## Conclusions

Exposure to serious adverse events especially malnutrition in early life might increase CVD mortality in later life. Improvements in medical services, pollution control, and lifestyle promotion could decrease CVD death. New strategy is needed to prevent CVD death and burden in the future.

## Supplementary information

**Additional file 1.**

## Data Availability

The data analysed in this study can be accessed from the vital registration system of health committee, Shanghai, China.

## Abbreviations

AAPC: Average annual percentage change
APC: Age-period-cohort
AYLL: Average years of life lost
CVD: Cardiovascular disease
CI: Confidence interval
ICD: International Classification of Disease
IHD: Ischemic heart disease
HD: Hypertensive disease
HS: Hemorrhagic stroke
IS: Ischemic stroke
LMICs: Low- and middle-income countries
PYLL: Person years of life lost
RHD: Rheumatic heart disease
SCD: Sequelae of cerebrovascular disease
SDI: Sociodemographic Index
SPYLLs: Age-standardized person years of life loss per 100,000 persons
