# Supplementary material for: Associations of socioeconomic factors with cause-specific Mortality and burden of cardiovascular diseases: findings from the vital registration in urban Shanghai, China, during 1974–2015
Source: BMC Public Health. 2020 Aug 26;20:1291. doi: 10.1186/s12889-020-09390-1 (PMC7448450; doi:10.1186/s12889-020-09390-1)
Supplement: Supplementary file 1 — Additional file 1. [file 12889_2020_9390_MOESM1_ESM.doc]

**Additional file**

**Associations of Socioeconomic Factors with Cause-specific Mortality and Burden of Cardiovascular Diseases: Findings from the Vital Registration in Urban Shanghai, China, during 1974-2015**

Lijuan Zhang^1,*^, Qi Li^1,*^, Xue Han^2,*^, Shuo Wang^3*^, Peng Li^4*^, Yibo Ding^4^, Tao Zhang^2^, Jia Zhao^2^, Yifan Chen^4^, Jiluo Liu^4^, Jue Li^1^, Rong Zhang^2^, Guangwen Cao^1,4^

**Correspondence**

Prof. Guangwen Cao

Department of Epidemiology, Faculty of Navy Medicine

Navy Medical University

8 Panshan Rd., Yangpu District, Shanghai 200433, People’s Republic of China

Tel & Fax: +86-21-8187-1060

Email: caoguangwen1965@163.com

**Table S1.** List of International Classification of Diseases (ICD) codes mapped to the Global Burden of Disease cause list for causes of cardiovascular disease death.

**Table S2**. The average life expectancy in Shanghai, China, 1974-2015.

**Figure S1.** The major causes of cardiovascular disease (CVD) death in Yangpu district, Shanghai, China, 1974- 2015.

**Table S3.** Trends in age-standardized mortality rates: 1974-2015.

**Table S4.** The association between age-standardized mortality rates for major causes of Carciovescular disease (CVD) and Sociodemographic Index (SDI) in Yangpu district, Shanghai, China, 1974- 2015.

**Table S5.** Trends in crude person years of life lost of cardiovascular disease (CVD) in Yangpu district, Shanghai, China, 1974-2015.

**Table S1. List of International Classification of Diseases (ICD) codes mapped to the Global Burden of Disease cause list for causes of cardiovascular disease death**

| **Cause** | **ICD-9** | **ICD-10** |
| --- | --- | --- |
| Cardiovascular diseases | 212.7, 225.0-225.9, 228.0-228.1, 391-391.9, 392.0, 393-398.9, 401-405, 410-414.9, 417-417.9, 420- 429.0, 430-436, 437.0-437.2,437.5-437.8, 438, 440.2, 440.4, 441-443.9, 447-454.9, 456, 456.3-457,457.1, 457.8-457.9, 459.1-459.3 | I01-I02.0, I05-I99, D15.1, D18.0-D18.1, D32.0-D32.9, D33.0-D33.2 |
| Ischemic heart disease | 410-414.9 | I20-I25.9 |
| Hemorrhagic stroke | 430-432.9, 437.2 | I60-I62.9, I67.0-I67.1, I68.1-I68.2 |
| Ischemic stroke | 433-435.9, 437.0-437.1, 437.5-437.8 | G45-G46.8, I63-I63.9, I65-I66.9, I67.2-I67.3, I67.5-I67.6 |
| Sequelae of cerebrovascular disease | 438 | I69.0-I69.8 |
| Hypertensive disease | 401-405.9 | I10-I15 |
| Rheumatic heart disease | 391-391.9, 392.0, 393-398.9 | I01-I01.9, I02.0, I05-I09.9 |
| Other forms of heart disease | 420-429 | I30-I52.8 |
| Other cardiovascular and circulatory diseases | 212.7, 225.0-225.9, 228.0-228.1, 393-398.9, 417-417.9, 420- 429.0, 437.0-437.2,437.5-437.8, 440.2, 440.4, 441-443.9, 447-454.9, 456, 456.3-457,457.1, 457.8-457.9, 459.1-459.3 | D15.1, D18.0-D18.1, D32.0-D32.9, D33.0-D33.2, I26-I28.9, I64, I67.1, I67.4, I67.7-I67.9, I68.0, I68.8, I70-I99 |

**Table S2.** **The average life expectancy in Shanghai, China, 1974-2015**

| **Years** | **All** | **Women** | **Men** |
| --- | --- | --- | --- |
| *1974* | 71.51 | 73.27 | 69.02 |
| *1975* | 71.61 | 73.47 | 69.13 |
| *1976* | 71.71 | 73.66 | 69.24 |
| *1977* | 72.53 | 74.22 | 69.97 |
| *1978* | 73.35 | 74.78 | 70.69 |
| *1979* | 73.14 | 75.48 | 70.64 |
| *1980* | 73.33 | 75.36 | 71.25 |
| *1981* | 73.38 | 75.47 | 71.28 |
| *1982* | 74.04 | 76.25 | 71.77 |
| *1983* | 73.23 | 75.26 | 71.15 |
| *1984* | 73.9 | 76.17 | 71.93 |
| *1985* | 74.27 | 76.37 | 72.14 |
| *1986* | 74.71 | 76.85 | 72.54 |
| *1987* | 74.46 | 76.6 | 72.32 |
| *1988* | 74.46 | 76.77 | 72.5 |
| *1989* | 74.98 | 77.12 | 72.85 |
| *1990* | 75.46 | 77.74 | 73.16 |
| *1991* | 75.79 | 77.74 | 73.58 |
| *1992* | 75.97 | 77.91 | 74.04 |
| *1993* | 75.97 | 77.91 | 74.04 |
| *1994* | 76.26 | 78.23 | 74.29 |
| *1995* | 76.03 | 77.97 | 74.11 |
| *1996* | 76.11 | 77.97 | 74.11 |
| *1997* | 77.2 | 79.21 | 75.18 |
| *1998* | 77.03 | 79.02 | 75.06 |
| *1999* | 78.44 | 80.53 | 76.38 |
| *2000* | 78.77 | 80.81 | 76.71 |
| *2001* | 79.66 | 81.83 | 77.47 |
| *2002* | 79.52 | 81.63 | 77.36 |
| *2003* | 79.8 | 81.83 | 77.78 |
| *2004* | 80.29 | 82.48 | 78.08 |
| *2005* | 80.13 | 82.36 | 77.89 |
| *2006* | 80.97 | 83.29 | 78.64 |
| *2007* | 81.08 | 83.29 | 78.87 |
| *2008* | 81.28 | 83.5 | 79.06 |
| *2009* | 81.73 | 84.06 | 79.42 |
| *2010* | 82.13 | 84.44 | 79.82 |
| *2011* | 82.51 | 84.8 | 80.23 |
| *2012* | 82.41 | 84.67 | 80.18 |
| *2013* | 82.47 | 84.79 | 80.19 |
| *2014* | 82.29 | 84.59 | 80.04 |
| *2015* | 82.75 | 85.09 | 80.47 |


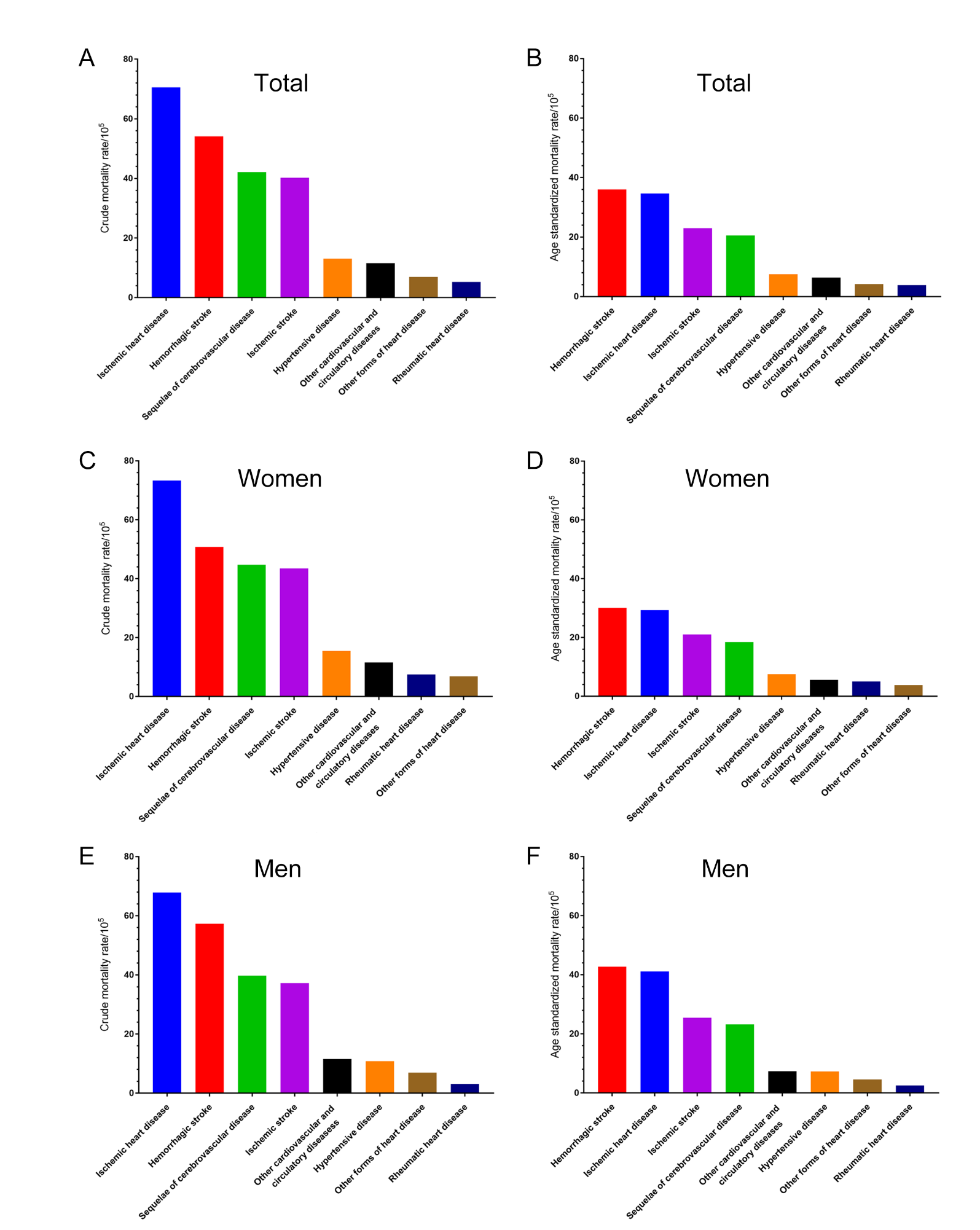


**Figure S1. The major causes of cardiovascular disease (CVD) death in Yangpu, Shanghai, China, 1974- 2015**

(A) crude mortality rates of total population; (B) age-standardized mortality rates of total population;

(C) crude mortality rates of female population; (D) age-standardized mortality rates of female population;

(E) crude mortality rates of male population; (F) age-standardized mortality rates of male population.

Other forms of heart disease include heart failure, pericarditis and other diseases of pericardium, acute and subacute endocarditis, heart valve diseases, myocarditis and cardiomyopathy, conduction diseases, cardiac arrest, cardiac arrhythmias.

**Table S3. Trends in the age-standardized mortality rates, in Yangpu, Shanghai, China,** 1974-2015

|  |  | **Joinpoint trend 1** | | **Joinpoint trend 2** | | **Joinpoint trend 3** | |
| --- | --- | --- | --- | --- | --- | --- | --- |
| **Disease** | **AAPC (95%CI) (1974-2015)** | **Years** | **AAPC (95%CI)** | **Years** | **AAPC (95%CI)** | **Years** | **AAPC (95%CI)** |
| **Total CVD** | -1.0 (-1.7 to -0.2)* | 1974-1998 | 0.8 (0.3 to 1.2)* | 1998-2003 | -9.2 (-14.0 to -4.1)* | 2003-2015 | -0.8 (-1.8 to 0.2) |
| Women | -1.3 (-2.0 to -0.7)* | 1974-1997 | 0.6 (0.2 to 1.1)* | 1997-2004 | -7.4 (-10.2 to -4.5)* | 2004-2015 | -1.4 (-2.6 to -0.2)* |
| Men | -0.7 (-1.5 to 0.1) | 1974-1998 | 1.1 (0.5 to 1.6)* | 1998-2003 | -9.5 (-14.8 to -3.9)* | 2003-2015 | -0.3 (-1.3 to 0.7) |
| **Ischemic stroke** | -2.5 (-3.1 to -1.8)* | 1974-2015 | -2.5 (-3.1 to -1.8)* |  |  |  |  |
| Women | -2.9 (-3.5 to -2.3)* | 1974-2015 | -2.9 (-3.5 to -2.3)* |  |  |  |  |
| Men | -2.2 (-2.8 to -1.5)* | 1974-2015 | -2.2 (-2.8 to -1.5)* |  |  |  |  |
| **Hemorrhagic stroke** | -3.2 (-4.3 to -2.1)* | 1974-1995 | 0.9 (0.1 to 1.7)* | 1995-2003 | -14.5 (-18.1 to -10.8)* | 2003-2015 | -2.4 (-4.7 to 0.0) |
| Women | -4.0 (-5.6 to -2.4)* | 1974-1995 | 0.7 (-0.4 to 1.8) | 1995-2003 | -15.7 (-20.6 to -10.5)* | 2003-2015 | -3.8 (-7.2 to -0.2)* |
| Men | -2.8 (-3.7 to -1.9)* | 1974-1995 | 1.1 (0.4 to 1.8)* | 1995-2003 | -14.0 (-17.0 to -10.9)* | 2003-2015 | -1.5 (-3.4 to 0.4) |
| **Ischemic heart disease** | 1.3 (0.3 to 2.3)* | 1974-1984 | 1.7 (-1.4 to 5.0) | 1984-1995 | 6.3 (4.0 to 8.6)* | 1995-2015 | -1.6 (-2.1 to -1.1)* |
| Women | 2.0 (1.4 to 2.6)* | 1974-1995 | 5.3 (4.2 to 6.5)* | 1995-2015 | -1.4 (-2.0 to -0.8)* |  |  |
| Men | 1.8 (1.0 to 2.6)* | 1974-1996 | 4.5 (3.5 to 5.4)* | 1996-2011 | -2.6 (-3.6 to -1.6)* | 2011-2015 | 3.9 (-1.9 to 10.0) |
| **Sequelae of cerebrovascular**  **disease** | 1.2 (-0.8 to 3.1) | 1974-1999 | 3.2 (1.9 to 4.6)* | 1999-2004 | -11.3 (-22.9 to 2.2) | 2004-2015 | 2.5 (-0.3 to 5.3) |
| Women | 0.6 (-1.4 to 2.7) | 1974-1999 | 2.6 (1.3 to 3.9)* | 1999-2004 | -11.0 (-23.3 to 3.3) | 2004-2015 | 1.8 (-1.1 to 4.9) |
| Men | 1.7 (-1.0 to 4.3) | 1974-1999 | 4.2 (2.7 to 5.9)* | 1999-2003 | -14.8 (-33.5 to 9.2) | 2003-2015 | 2.3 (-0.3 to 5.0) |
| **Rheumatic heart disease** | -6.0 (-7.1 to -4.9)* | 1974-1979 | -7.6 (-14.1 to -0.7)* | 1979-1990 | 1.6 (-0.9 to 4.2) | 1990-2015 | -8.8 (-9.6 to -8.1)* |
| Women | -5.5 (-6.5 to -4.5)* | 1974-1991 | -0.4 (-2.1 to 1.3) | 1991-2015 | -9.0 (-10.3 to -7.7)* |  |  |
| Men | -5.8 (-7.0 to -4.7) | 1974-1993 | -1.9 (-3.5 to -0.2)* | 1993-2015 | -9.2 (-10.9 to -7.4)* |  |  |
| **Hypertensive disease** | -0.8 (-2.3 to 0.7) | 1974-1978 | 7.0 (-7.1 to 23.2) | 1978-2003 | -3.6 (-4.4 to -2.8)* | 2003-2015 | 2.6 (0.7 to 4.6)* |
| Women | -1.1 (-2.8 to 0.7) | 1974-1978 | 11.4 (-5.4 to 31.1) | 1978-2002 | -4.3 (-5.3 to -3.3)* | 2002-2015 | 1.3 (-0.8 to 3.5) |
| Men | -0.7 (-1.4 to 0.1) | 1974-2007 | -2.2 (-2.8 to -1.7)* | 2007-2015 | 6.0 (2.5 to 9.6)* |  |  |
| **Other forms of heart disease** | 0.3 (-1.1 to 1.6) | 1974-1990 | -3.4 (-6.3 to -0.4)* | 1990-2015 | 2.7 (1.5 to 3.8)* |  |  |
| Women | -0.7 (-1.5 to 0.1) | 1974-2015 | -0.7 (-1.5 to 0.1) |  |  |  |  |
| Men | 1.1 (-0.7 to 3.0) | 1974-1987 | -4.7 (-9.8 to 0.8) | 1987-2015 | 3.9 (2.9 to 5.0)* |  |  |
| **Other cardiovascular and**  **circulatory diseases** | -3.3 (-6.3 to -0.2) | 1974-2000 | -6.8 (-9.8 to -3.6)* | 2000-2015 | 3.1 (-3.6 to 10.3) |  |  |
| Women | -4.0 (-5.5 to -2.5)* |  |  |  |  |  |  |
| Men | -3.0 (-6.0 to 0) | 1974-2000 | -7.2 (-10.4 to -4.0)* | 2000-2015 | 4.7 (-1.8 to 11.7) |  |  |

Other forms of heart disease include heart failure, pericarditis and other diseases of pericardium, acute and subacute endocarditis, heart valve diseases, myocarditis and cardiomyopathy, conduction diseases, cardiac arrest, cardiac arrhythmias; AAPC: average annual percentage change; UI: 95% uncertainty intervals; *: AAPC value is significantly different from 0 at α=0.05.

**Table S4. The association between the age-standardized mortality rates for major causes of CVD and SDI in Yangpu, Shanghai, China, 1974- 2015**

| **Causes of CVD death** | **Regression coefficients, *β*** | ***t*** | ***P* value** |
| --- | --- | --- | --- |
| Total | -0.690 | -6.028 | 4.3×10^-7^ |
| Rheumatic heart disease | -0.927 | -15.685 | 1.1×10^-18^ |
| Ischemic heart diseases | 0.646 | 5.354 | 4.0×10^-5^ |
| Hemorrhagic stroke | -0.796 | -8.318 | 3.0×10^-10^ |
| Ischemic stroke | -0.684 | -5.929 | 5.9×10^-7^ |
| Hypertensive disease | -0.781 | -7.902 | 1.1×10^-9^ |
| Sequelae of cerebrovascular disease | 0.222 | 1.443 | 0.157 |
| Other forms of heart diseases | 0.289 | 1.912 | 0.063 |
| Other cardiovascular and circulatory diseases | -0.125 | 0.796 | 0.431 |

The P-value indicates the association of the age-standardized mortality rates for major causes of cardiovascular disease (CVD) with sociodemographic index (SDI), the *β* coefficient indicates the strength of association.

**Table S5. Trends in crude person years of life lose for cardiovascular disease (CVD) in Yangpu, Shanghai, China,** 1974-2015

|  |  | **Joinpoint trend 1** | | **Joinpoint trend 2** | | **Joinpoint trend 3** | |
| --- | --- | --- | --- | --- | --- | --- | --- |
| **Disease** | **AAPC**  **(95% UI)**  **(1974-2015)** | **Years** | **AAPC**  **(95% UI)** | **Years** | **AAPC**  **(95% UI)** | **Years** | **AAPC**  **(95% UI)** |
| Ischemic heart disease | 1.6 (1.2 to 2.1)* |  |  |  |  |  |  |
| Hemorrhagic stroke | 0.8 (-0.1 to 1.7) | 1974-1987 | 6.1 (4.5 to 7.8)* | 1987-2007 | -3.3 (-4.2 to -2.4)* | 2007-2015 | 2.9 (-0.4 to 6.2) |
| Ischemic stroke | 2.1 (-4.1 to 8.6) |  |  |  |  |  |  |
| Sequelae of cerebrovascular disease | 5.1 (-3.0 to 13.9) | 1974-1977 | 66.9 (-0.8 to 180.7) | 1977-1980 | -22.8 (-71.7 to 110.4) | 1980-2015 | 3.8 (2.5 to 5.1)* |
| Hypertensive diseases | 2.5 (0.3 to 4.9)* | 1974-1977 | 6.0 (-19.1 to 38.9) | 1974-1994 | -3.3 (-5.6 to -1.1)* | 1994-2015 | 7.1 (5.4 to 8.8)* |
| Rheumatic heart disease | -5.6 (-7.4 to -3.7)* | 1974-1982 | -8.3 (-12.5 to -2.8)* | 1982-1989 | 8.4 (-0.9 to 18.5) | 1989-2015 | -8.2 (-9.6 to -6.9)* |
| Other forms of heart disease | 3.1 (1.4 to 4.8)* | 1974-1982 | -4.7 (-11.9 to 3.1) | 1982-2015 | 5.1 (4.2 to 6.0)* |  |  |
| Other cardiovascular and circulatory diseases | 0.7 (-9.2 to 11.8) | 1974-1990 | 3.2 (-2.8 to 9.5) | 1990-1993 | 62.1 (-60.7 to 568.8) | 1993-2015 | -7.2 (-10.6 to -3.7)* |

Other forms of heart disease include heart failure, pericarditis and other diseases of pericardium, acute and subacute endocarditis, heart valve diseases, myocarditis and cardiomyopathy, conduction diseases, cardiac arrest, cardiac arrhythmias; AAPC, average annual percentage change; CVD, cardiovascular disease; UI, uncertainty intervals; *: AAPC value is significantly different from 0 at α=0.05.


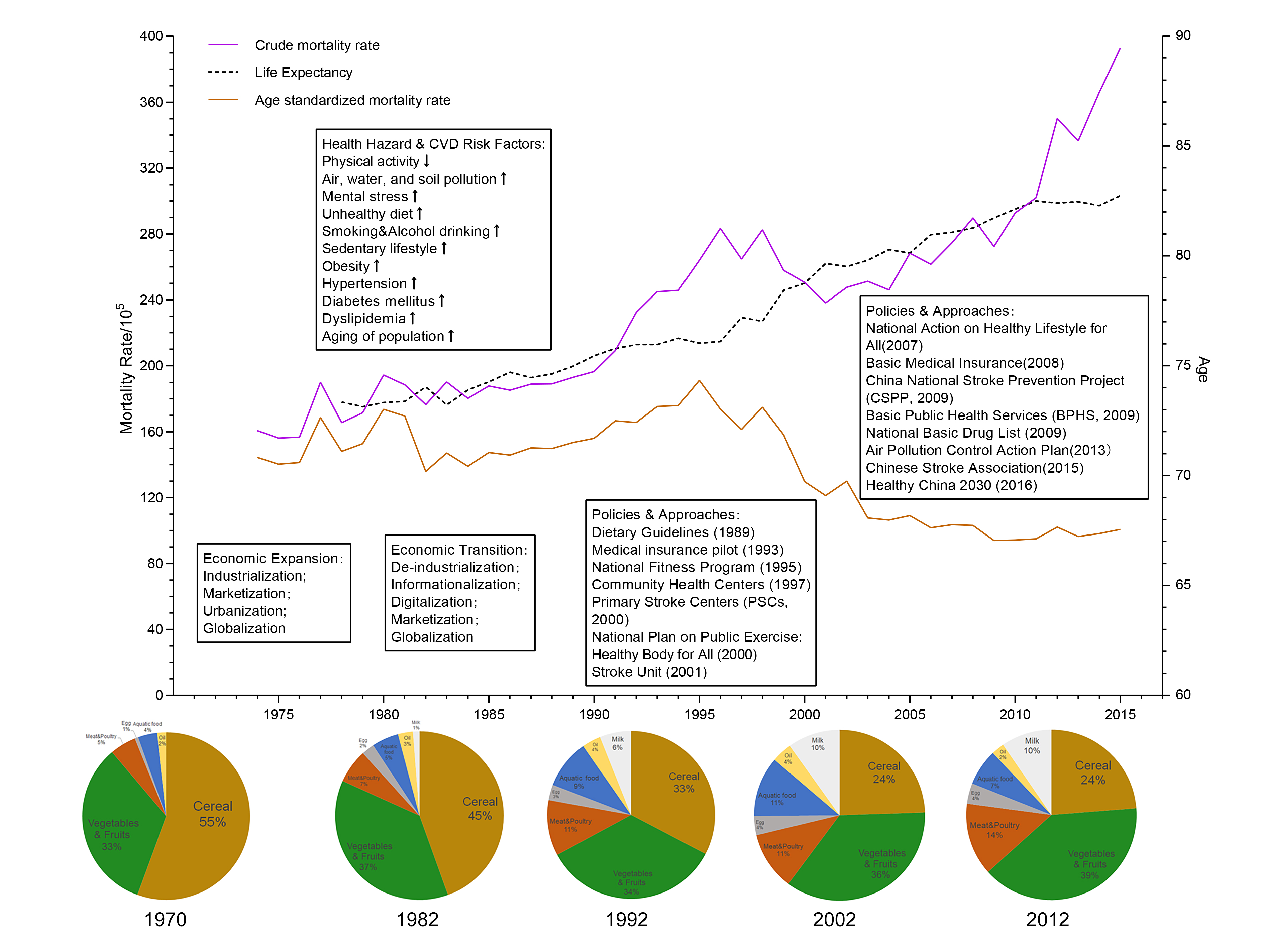


**Figure S2. Rates of crude mortality and age-standardized mortality of cardiovescular disease (CVD) and socioeconomic events and change in dietary patterns in Yangpu, Shanghai, China from 1974 to 2015.** The policies issued at the different timepoints were obtained from Center for Disease Control and Prevention of Yangpu, Shanghai, China. The information on dietary patterns in urban Shanghai was obtained from a Shanghai Center for Disease Control database, as previously described [21].
